# The perils of contact sport: pathologies of diffuse brain swelling and chronic traumatic encephalopathy neuropathologic change in a 23-year-old rugby union player

**DOI:** 10.1007/s00401-023-02576-y

**Published:** 2023-04-22

**Authors:** Edward B. Lee, Claire Kennedy-Dietrich, Jennian F. Geddes, James A. R. Nicoll, Tamas Revesz, Douglas H. Smith, William Stewart

**Affiliations:** 1 Translational Neuropathology Research Laboratory, University of Pennsylvania, Philadelphia, PA 19104 USA; 2 School of Psychology and Neuroscience, University of Glasgow, Glasgow, G12 8QQ UK; 3 (Formerly) Queen Mary, University of London, London, UK; 4 Clinical Neurosciences, University of Southampton, Southampton, SO16 6YD UK; 5 Queen Square Brain Bank for Neurological Disorders, UCL Queen Square Institute of Neurology, University College London, London, WC1N 3BG UK; 6 Department of Neurosurgery, Penn Center for Brain Injury and Repair, Perelman School of Medicine, University of Pennsylvania, Philadelphia, PA 19104 USA; 7 Department of Neuropathology, Queen Elizabeth University Hospital, 1345 Govan Rd, Glasgow, G51 4TF UK

There is increasing awareness of the consequences of traumatic brain injury (TBI) and repetitive head impact exposure (RHI) in contact sport, largely driven by greater recognition of chronic traumatic encephalopathy neuropathologic change (CTE-NC) [6, 12, 13]. Nevertheless, prior to consensus criteria for the neuropathological assessment of CTE-NC [1, 9], there were isolated cases and short case series documenting neurodegenerative pathologies in former contact sports athletes. Included among these were reports of hyperphosphorylated tau pathologies suggestive of CTE-NC in autopsy studies of relatively young individuals with RHI exposures [4]. Here we re-examine one such example, Case 5 in Geddes et al., 1999 [4], employing current neuropathological consensus criteria and informed by clinical history obtained via contemporary next of kin interview.

A 23-year-old, male, elite rugby union player was knocked unconscious while tackling an opponent. He was attended to on the field, rapidly regained consciousness and returned to the match in the next play before reporting symptoms of headache, collapsing, and becoming unresponsive. He was immediately transferred to hospital, where he was breathing spontaneously, with a Glasgow Coma Scale of 4 out of 15, a pulse of 120 beats per minute and blood pressure 206/120 mmHg. His pupils were fixed and dilated and he had bilateral up-going plantars. CT scan demonstrated loss of gray/ white matter definition, with a small right-sided subdural hematoma (SDH). Intracranial pressures were recorded at 90 mmHg. He was managed conservatively but continued to decline to his death just over 24 h from injury.

His history was remarkable for frequent rugby-associated head injuries, estimated up to 3 concussions with loss of consciousness per season. Most notably, shortly prior to this admission, he sustained a concussion with brief (‘seconds’) loss of consciousness, leading to his removal from the field of play. He reported no symptoms following the match, returned to training as normal in the days thereafter and played his next competitive game 10 days later (his final match). He had no history of health problems, was a postgraduate student and reported no history of altered behavior or mental health disorder. He was a keen athlete, but never participated in combat sports or soccer, drank socially, was a non-smoker and there was no known history of non-prescribed drug use or TBI outside of rugby.

At autopsy, thin film, right convexity SDH was confirmed (approximately 10 ml). Evidence of diffuse brain swelling was present, without measurable midline shift. However, there was evidence of internal herniation with displacement of the medial temporal lobes and cerebellar tonsils and central pontine hemorrhage. Cavum septum pellucidum was present (up to 2 mm anteriorly; Fig. 1a). The original diagnostic histological sections were reviewed, to which were added additional tissue samples and immunocytochemistry for amyloid precursor protein (22C11; APP) and for hyperphosphorylated tau (p-tau; PHF-1), amyloid-beta (6f3d), alpha-synuclein (KM51), and phosphorylated TDP-43 (1D3), in line with consensus criteria for evaluation of CTE-NC [1, 9].

**Fig. 1:**
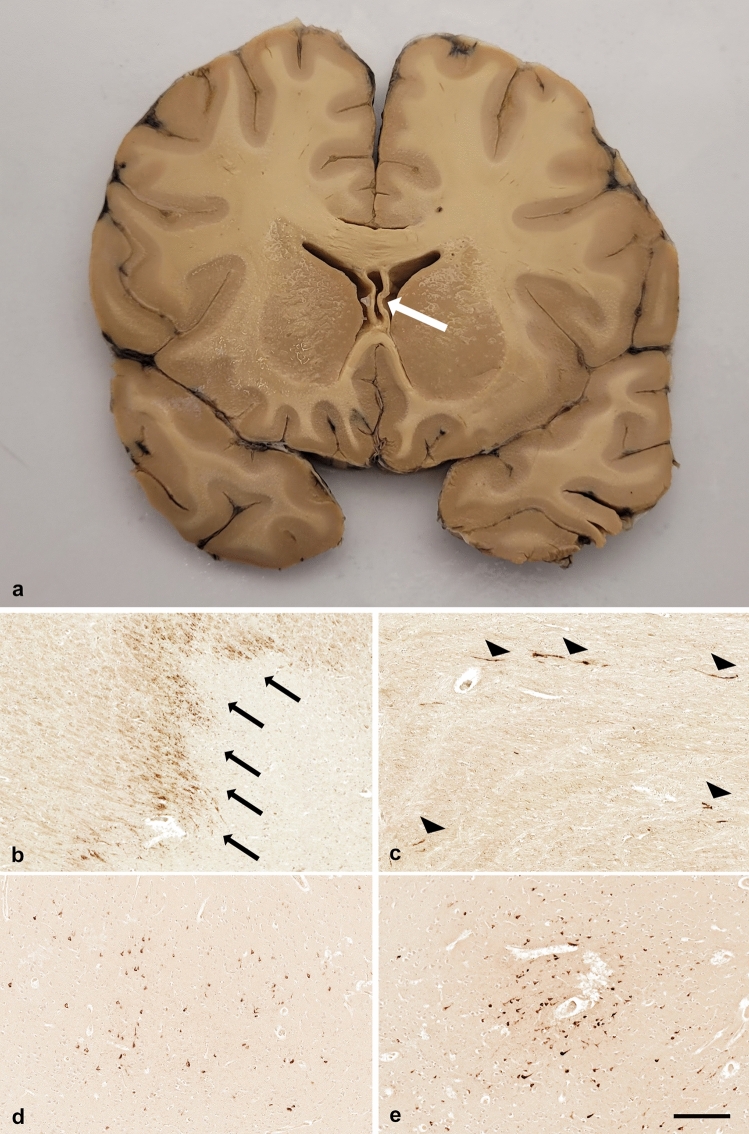
**a** Examination of the brain revealed evidence of diffuse brain swelling, with ventricular effacement but no measurable midline shift. Cavum septum pellucidum was present (white arrow). On microscopy, waves of punctate to granular APP-immunoreactive axons were present consistent with the vascular complications of raised intracranial pressure (**b**, arrows), in addition to scattered linear and fusiform axonal profiles with the stereotypical appearance and distribution of DAI (**c**, arrowheads). Multiple patchy foci of p-tau-immunoreactive neuronal profiles were present within the neocortex, typically at sulcal depths (**d**) with, in several locations, perivascular clustering of p-tau-immunoreactive neurons consistent with the pathognomonic lesion of CTE-NC (**e**). Scale bar 250 µm **b**–**e**

Sections stained for APP revealed immunoreactive axons in two distinct patterns. In and around the deep gray nuclei and surrounding the hemorrhagic focus in the pons, waves of APP-immunoreactive axons typical of the vascular complications of raised intracranial pressure were present (Fig. 1b) [10]. Elsewhere, within the corpus callosum and internal capsule, frequent, scattered, linear, and fusiform APP-immunoreactive axons were present, in keeping with diffuse traumatic axonal injury **(**DAI; Fig. 1c) [5]. Staining for p-tau revealed numerous foci of neurofibrillary tangles and scattered neurites, typically in mid-cortical layers at the depths of sulci and adjacent sulcal margins, and extending to both superficial and, less so, deeper layers (Fig. 1d). In multiple locations, these showed stereotypical, pathognomonic appearances of CTE-NC, including perivascular clustering (Fig. 1e). Occasional p-tau immunoreactive neurons were identified in the amygdala, consistent with low-stage CTE-NC [1]. No other supportive neuronal or astroglial p-tau pathologies were observed. Stains for amyloid-beta, alpha-synuclein, and phosphorylated TDP-43 were negative.

Regarding acute TBI, the clinical presentation and neuropathology were of rapid-onset, severe, diffuse brain swelling with small volume SDH, insignificant midline shift and DAI which, in context of the history of mild TBI 10 days prior to this event, might be interpreted as consistent with second impact syndrome [2, 14]. Rapid, diffuse brain swelling leading to fatal outcome is an exceptionally rare event following mild TBI. Typically encountered in adolescents and young adults, often there is history of injury in the preceding days, hence the commonly used term second impact syndrome [2]. However, whether recent TBI is necessary or simply coincident is uncertain [3]. Nevertheless, sports concussion management is intended to mitigate risk of such adverse outcomes, with immediate removal from play for any suspected concussion, followed by a graduated return to sport [7].

Over and above acute TBI pathology, examination informed by current consensus criteria revealed multiple foci of p-tau immunoreactive neuronal profiles in stereotypical pattern and distribution of CTE-NC [1, 9]. Notably, this pathology was not observed in the original, thorough, diagnostic work-up and research reporting of this case [4]. As such, this re-appraisal of available archival materials underlines the utility of published consensus criteria for the neuropathological evaluation of CTE. To date, the only documented risk factor for CTE-NC is exposure to RHI or a single moderate or severe TBI [9, 12]. Long recognized as a neurodegenerative disease associated with boxers, our and others’ experience from autopsy studies demonstrates CTE-NC present in around 75% of former contact sports athletes with dementia [6], with epidemiological data revealing high neurodegenerative disease risk among former elite rugby players [11].

There have been only isolated autopsy studies of young athletes reporting evidence of CTE-NC [8]. To date, however, none with RHI/TBI exclusively through rugby union. This observation provides evidence of established neurodegenerative pathology in early life, active, contact sports participants, in advance of any recognizable clinical manifestation. Detection of CTE-NC at such young age supports the hypothesis that the processes driving TBI-related neurodegeneration are established many years prior to clinical presentation, reinforcing calls to limit exposure to sports-associated RHI and TBI.

## Data Availability

The data supporting the conclusions of this article are included within the article.
